# Pasteur’s Precautions against the Cholera

**Published:** 1883-10

**Authors:** 


### Pasteur's Precautions Against the Cholera.

M. Pasteur has given the following instructions to the members of the mission sent by the French government to study the present epidemic of cholera. These instructions all relate to the contingency of resisting the maximum amount of causes of contagion, and are framed on the hypothesis that cholera does not enter the animal economy by the respiratory passages, but by the digestive tract only, except in very exceptional cases

They are contained in the *Revue de Hygiene* for August, 1883. 1. The drinking-water of the locality where the mission settles to undertake researches should not be used without having been first boiled, and shaken, when cold, for two or three minutes in a phial or bottle, half filled and stoppered. The water of the locality may be used if it be drawn at the source itself in vessels which have been exposed some moments to air heated to about 150° Cent. (302° Fahr.), or, better still, to a higher temperature. Natural mineral waters may be used with advantage. 2. Wine which has been warmed in bottles to from 55° to 60° Cent. (131° to 140° Fahr.), may be drunk in glasses which have been warmed in a similar way. 3. Thoroughly cooked food only should be used, or raw fruit well washed in water which has been boiled, and which has been kept in the same vessels in which it was boiled, or which has been poured from those vessels into other heated vessels. 4. Bread should be cut into thin slices and exposed to a heat of about 150° Cent. for twenty minutes at the utmost after it has been cut in slices. 5. All the vessels employed in the preparation of food should be warmed to a temperature of 150° or more. 6. Bed-clothes and body-linen should be plunged into boiling water and then dried. 7. Water for washing should be brought to boiling point; and, after cooling, one five-hundredth part of thymic acid (a litre, 1¾ pint, of alcoholized water to two grammes of acid), should be added, or one-fiftieth (a litre of water to twenty grammes) of carbolic acid. 8. The face and hands should be washed several times daily with water which has been boiled, and to which carbolic acid dissolved in water has been added. 9. In cases only when it would be required to handle the bodies of cholera patients, or sheets or body-linen soiled with their injections, it would be necessary to cover the mouth and the nostrils with a little mask formed of two pieces of fine metallic cloth, containing between their surfaces wadding about the thickness of one centimètre or more. This mask should be warmed to 150°, and the temperature should be renewed on each fresh occasion of peculiar infection.

